# DIFFERENCES IN RESILIENCE AMONG MIDLIFE AND OLDER ADULTS BY SEXUAL AND GENDER IDENTITY AND BY GENERATION

**DOI:** 10.1093/geroni/igad104.3280

**Published:** 2023-12-21

**Authors:** Christi Nelson, Karen Fredriksen-Goldsen

**Affiliations:** University of Washington, Seattle, Washington, United States; University of Washington, Seattle, Washington, United States

## Abstract

Sexual and gender minority (SGM) (i.e., lesbian, gay, bisexual, sexual diverse, and transgender) midlife and older adults face specific health, social, and structural inequities in addition to the late-life challenges experienced by older adults in general. Extant research had largely examined health disparities and related risk and protective factors among SGM individuals as a homogeneous group, without attending to the heterogeneity among the subgroups and differing generations. Most research has also focused on stress with limited attention to resilience, even though higher resilience is associated with better health when facing adversity. Using baseline data from Aging with Pride: The National Health, Aging, and Sexuality/Gender Study, we identify historical/environmental, psychological, and social risk and protective factors that are associated with resilience in midlife and older SGM adults (n=2,450), aged 50-102, and examine differences in the associations between the factors and resilience by SGM and generational subgroups (i.e., Invisible, Silenced, and Pride). The results revealed differences in significant factors associated with resilience by SGM and generational subgroups. Identity affirmation was positively associated with resilience in all but the bisexual and transgender groups. Day-to-day discrimination was negatively associated with resilience for all but bisexual women. Community belongingness was positively associated with resilience for gay and bisexual men and the younger generations (Silenced and Pride). These results suggest that factors associated with fostering resilience, and in turn better health, may differ by SGM subgroup as well as by generation. Assessing differences in factors that promote resilience can help develop responsive interventions for targeted subgroups.

